# A Case Report of Acute Renal Failure due to Amlodipine Overdose and Alcohol Consumption

**DOI:** 10.1002/ccr3.72529

**Published:** 2026-04-15

**Authors:** Yi Xi

**Affiliations:** ^1^ Department of Nephrology, Wusong Branch Zhongshan Hospital Affiliated to Fudan University Shanghai China

**Keywords:** acute kidney injury (AKI), acute tubular necrosis (ATN), alcohol consumption, amlodipine overdose

## Abstract

Amlodipine intoxication is clinically common yet life‐threatening, usually resulting in severe cardiovascular collapse and end‐organ hypoperfusion. Here, we report a case of a 46‐year‐old male with amlodipine (420 mg) and ethanol intoxication who unexpectedly presented without severe hypotension or shock. Instead, his main manifestation was acute renal function loss requiring hemodialysis. The potential pathological mechanism may be acute kidney injury (AKI) caused by transient renal ischemic insult, while ethanol may exacerbate acute tubular necrosis (ATN).

## Key Clinical Message

Amlodipine overdose can cause severe acute kidney injury requiring dialysis even in the absence of profound hypotension. Concomitant alcohol ingestion may exacerbate renal tubular injury. Early recognition and close renal monitoring are essential, even when initial hemodynamic parameters appear relatively stable.

## 1 Introduction

Amlodipine, a classic dihydropyridine calcium channel blocker, is the most widely prescribed antihypertensive agent due to its slow and long duration of action [1]. In some regions, patients can easily obtain it without a clinician's prescription. Globally, the incidence of amlodipine overdose has increased significantly in recent years, primarily due to its convenience and easy accessibility, and it has become the leading cause of deaths associated with cardiovascular medication exposure [2]. Amlodipine poisoning can be lethal even at a moderate dose, which is characterized by severe cardiovascular collapse and end‐organ hypoperfusion, including refractory shock, pulmonary edema, renal failure, and bradycardia or tachycardia. Additionally, blockade of calcium channels in pancreatic beta cells can lead to reduced insulin secretion [3]. However, current standard therapeutic modalities—including immediate gastrointestinal decontamination, high‐dose insulin therapy, calcium supplementation, vasopressors, lipid emulsion therapy, hemodiafiltration, and inotropic agents—remain inadequate [4, 5, 6]. Alcohol consumption is also one of the major global public health problems. Acute kidney injury (AKI) is a complication of severe alcohol‐associated hepatitis. Furthermore, several studies have indicated that alcohol consumption can directly impair glomerular filtration and renal tubular reabsorption processes [7, 8]. Here, we describe a case of a patient with amlodipine overdose and acute alcohol ingestion who presented to our hospital with relatively stable blood pressure but unexpectedly developed AKI and required hemodialysis.

## 2 Case History/Examination

A 46‐year‐old obese male patient with irregular antihypertensive treatment, a history of chronic alcohol abuse, and depressive disorder had a previous suicide attempt by ingesting eight tablets of zolpidem tartrate and an unspecified amount of wine. At that time, he went to the emergency department and underwent a basic physical examination, which showed normal vital signs, a normal electrocardiogram (ECG), and normal renal function (serum creatinine: 0.77 mg/dL, blood urea: 4.6 mmol/L). He received only fluid therapy and was advised to attend a psychological clinic for further management. However, three weeks later, he made another suicide attempt by ingesting 84 tablets of amlodipine (total dose: 420 mg) along with 400 g of 50% ethanol (alcohol). Forty‐eight hours after ingestion, he was admitted to the emergency department of our hospital under his family's company, with symptoms of oliguria (approximately 100 mL in the last 24 h), lower limbs edema, cough, and chest discomfort. Upon arrival, he was conscious and cooperative. His vital signs were as follows: blood pressure 106/58 mmHg (mean arterial pressure (MAP) 74 mmHg), heart rate 78 beats per minute (bpm), respiratory rate 22 breaths per minute, and oxygen saturation 95% at room air. Gastrointestinal decontamination was not performed due to the delayed presentation. He received intravenous fluid resuscitation and was transferred to the Department of Nephrology. Blood and urine concentrations of amlodipine and alcohol were not measured due to the lack of relevant detection equipment. Ambulatory vital sign monitoring showed that the highest blood pressure was 138/80 mmHg, the lowest was 112/60 mmHg, and the average MAP (mean arterial pressure) was 85 mmHg. Heart rate fluctuated between 65 and 78 bpm. Laboratory examinations revealed a normal complete blood count, N‐terminal pro‐brain natriuretic peptide (NT‐pro BNP) level, troponin level, and liver enzyme levels. Electrolyte and serum pH levels were within normal range: serum potassium 3.9 mmol/L (NV: 3.5–5.5 mmol/L), serum sodium 135 mmol/L (NV: 135–145 mmol/L), serum calcium 2.2 mmol/L (NV: 2.1–2.6 mmol/L), serum pH 7.39 (NV: 7.35–7.45). While significant renal dysfunction was noted with a serum creatinine level of 5.5 mg/dL and blood urea level of 13.2 mmol/L. Urine dipstick test results were as follows: specific gravity 1.030 (normal value [NV] 1.003–1.030); pH 5.0 (NV: 5.0–8.0); albumin ± (NV: absent); nitrites negative; ketones, glucose, hemoglobin, and leukocyte esterase were absent. Urinary sediment examination showed: leukocytes 24/μL (NV: 0–5/μL), no erythrocytes, crystals 1/μL (NV: 0–14/μL), and casts 16/μL (NV: 0–2/μL), including granular and hyaline casts. Initial ECG showed nonspecific ST‐T segment changes. Chest computed tomography (CT) scan revealed interstitial edema without pleural effusions. Renal ultrasound showed no obvious abnormalities, and echocardiogram was unremarkable.

## 3 Differential Diagnosis, Investigations, and Treatment

Acute renal injury (AKI) was diagnosed based on the following triad: acute onset oliguria, significantly elevated serum creatinine and urea levels, and abnormal urinalysis findings [9]. Particular attention was paid to distinguishing this case from BRASH syndrome (Bradycardia, Renal failure, Atrioventricular block, Shock, Hyperkalemia), which is characterized by the presence of bradycardia, renal dysfunction, atrioventricular block, shock, and hyperkalemia. The patient in this case presented with severe AKI but no cardiac conduction block or electrolyte abnormalities, which distinguishes it from classic BRASH syndrome.

This patient was given oxygen, intravenous fluid infusion, and furosemide, but remained anuric. Twenty‐four hours after admission, his serum creatinine level peaked at 6.5 mg/dL; therefore we initiated blood purification treatment, including hemodialysis combined with hemoperfusion, to alleviate fluid overload and remove amlodipine. He received intermittent dialysis on alternate days, with a blood flow rate of 200 mL/min, a dialysate flow rate of 500 mL/min, and each session lasting 4 h. Two weeks later, his urine output began to increase to 1000 mL/24 h; however, his serum creatinine level remained elevated at 5.5 mg/dL, and dialysis was suspended. We recommended that this patient undergo a renal needle biopsy to clarify the pathogenesis, but he declined the procedure. After another week of conventional treatments, his renal function gradually improved. His serum creatinine level decreased to 1.07 mg/dL and he was subsequently discharged from the hospital for further psychiatric intervention.

## 4 Discussion

Amlodipine exerts its toxic effects primarily by blocking calcium channels in vascular smooth muscle, leading to vasodilation, refractory hypotension, and subsequent renal hypoperfusion, which is the classic mechanism of amlodipine‐induced AKI [10]. In previous reported cases of amlodipine overdose, patients typically presented with refractory shock and severe hypotension, accompanied by impaired consciousness and target organ hypoperfusion, which required administration of high‐dose vasoactive agents including dopamine, norepinephrine and metaraminol [3, 11, 12]. Inconsistent with the typical hemodynamic disturbance‐dominated AKI caused by amlodipine poisoning, the patient in this case maintained relatively stable blood pressure during the entire hospitalization period despite ingesting a massive dose of amlodipine (420 mg)—far beyond the therapeutic dose (5–10 mg), which is sufficient to cause refractory hypotension. Notably, this patient presented to the hospital 48 h after ingestion, suggesting that the most critical hypotensive phase (the peak of amlodipine's effect) likely occurred outside the hospital and had resolved by the time of admission. Since the sensitivity of individual patients to a decrease in kidney perfusion is variable, some patients may develop AKI after just a few minutes of hypotension, while others can tolerate hours of renal ischemia without structural renal damage [13]. Thus, the stable blood pressure during the hospitalization period does not rule out the possibility of transient severe hypotension before admission. This transient ischemic insult to the renal cortex would cause ATP depletion in renal tubular epithelial cells, resulting in cell necrosis, sloughing, and cast formation—pathological features that were confirmed by the patient's urinary sediment examination (granular and hyaline casts), which are typical of acute tubular necrosis (ATN) [14, 15]. In addition, the patient had a history of long‐term poorly controlled hypertension, which provides another explanation for the transient hypotensive insult. In patients with chronic hypertension, renal autoregulation adapts to the persistently elevated blood pressure levels. A sudden acute drop in blood pressure, even if the absolute value falls within the normal range, can result in a relative hypotensive state and lead to insufficient renal perfusion. This relative hypotension, combined with the direct vasodilatory effect of amlodipine, further exacerbates renal hypoperfusion. In such cases, early recognition of pathogenic factors, close monitoring of hemodynamic parameters, comprehensive assessment of the patient's condition, and individualization of therapeutic strategies (e.g., active use of vasopressors and rational use of blood purification therapy) are crucial to improving renal perfusion, preventing the progression of renal ischemia and avoiding irreversible ATN.

The patient's acute alcohol overdose (400 g of 50% ethanol) and history of chronic alcohol abuse further exacerbated renal injury. Previous studies have shown that ethanol and its metabolite acetaldehyde can directly damage the integrity of renal tubular epithelial cell membranes, inhibit cellular energy metabolism, and induce oxidative stress in renal tissue, thereby aggravating tubular cell necrosis [7, 8]. Furthermore, acute alcohol intoxication can indirectly reduce renal perfusion by inhibiting the central nervous system and causing mild vasodilation, while chronic alcohol abuse can lead to malnutrition and hypoalbuminemia, both of which further impair renal function [8, 16].

The pathogenic mechanism of AKI in this case is likely the synergistic toxic effect of amlodipine and ethanol, rather than the single toxic effect of either agent. The combined effect of these two factors (transient ischemia and alcohol toxicity) leads to the rapid progression of AKI, which also explains why the patient was unresponsive to diuretic therapy and required renal replacement therapy.

This case has several limitations that should be acknowledged. First, due to the lack of relevant detection equipment, blood and urine concentrations of amlodipine and ethanol were not measured, which prevents quantitative confirmation of the correlation between the dose of the two agents and the severity of AKI, as well as verification of the peak concentration of amlodipine during the transient hypotensive phase. Second, the patient refused renal needle biopsy, so the exact pathological type of AKI could not be clarified, which limited the in‐depth exploration of the pathogenic mechanism.

## 5 Conclusion

This case describes a rare clinical presentation of acute renal failure induced by a massive amlodipine overdose (420 mg) combined with acute alcohol ingestion, characterized by AKI requiring hemodialysis but without the typical severe hypotension or shock seen in most amlodipine poisoning cases [2, 3, 7, 8]. The unique clinical manifestation of this case is closely related to the transient severe hypotension that likely occurred before hospital admission, the patient's long‐term poorly controlled hypertension, and the synergistic toxic effect of ethanol on renal tissue. Our findings indicate that transient renal hypoperfusion caused by amlodipine overdose, even in the absence of persistent hypotension during hospitalization, can lead to irreversible ATN. Moreover, acute alcohol overdose can further exacerbate renal injury by directly damaging renal tubular epithelial cells and indirectly reducing renal perfusion. These findings emphasize the need for clinical vigilance in patients with amlodipine and alcohol co‐ingestion (even with stable admission vital signs), also comprehensive psychiatric care for those with a history of suicide attempts, and stricter amlodipine accessibility regulations to reduce intentional overdose.

Given the limitations of this single case report, further prospective studies with larger sample sizes and more comprehensive detection of drug concentrations are needed to clarify the exact pathogenic mechanisms of AKI induced by the synergistic effect of amlodipine and ethanol, and to optimize the clinical management strategy for such cases.

## Author Contributions

**Yi Xi:** funding acquisition, investigation, methodology, project administration, writing – original draft, writing – review and editing.

## Funding

This work was supported by Science and Technology Innovation Plan Of Shanghai Science and Technology Commission, 2023‐E‐06.

## Ethics Statement

This study was granted an ethical exemption by the Ethics Committee of Wusong Branch, Zhongshan Hospital Affiliated to Fudan University. Written informed consent was obtained from the patient's legally authorized representative for publication of the case report.

## Conflicts of Interest

The author declares no conflicts of interest.

## Data Availability

The data that support the findings of this study are available from the corresponding author upon reasonable request.

## References

[1] J. J. Bai , W. L. Zhang , and L. Wang , “Analysis of Prescription and Rationality of Anti‐Hypertensive Medication Among Community Health Centers in Beijing,” Chinese Journal of Cardiology 49, no. 10 (2021): 993–999. 34674437 10.3760/cma.j.cn112148-20201231-01022

[2] D. D. Gummin , J. B. Mowry , and M. C. Beuhler , “A Nnual Report of the American Association of Poison Control Centers' National Poison Data System(NPDS):37th Annual Report,” Clinical Toxicology 58, no. 12 (2020): 1360–1541. 33305966 10.1080/15563650.2020.1834219

[3] F. Karbek Akarca , E. Akceylan , and S. Kıyan , “Treatment of Amlodipine Intoxication With Intravenous Lipid Emulsion Therapy: A Case Report and Review of the Literature,” Cardiovascular Toxicology 17, no. 4 (2017): 482–486. 28766181 10.1007/s12012-017-9421-3

[4] B. Mokhlesi , J. B. Leikin , P. Murray , and T. C. Corbridge , “Adult Toxicology in Critical Care: Part II: Specific Poisonings,” Chest 123, no. 3 (2003): 897–922. 12628894 10.1378/chest.123.3.897

[5] T. A. Siddiqi , J. Hill , Y. Huckleberry , and S. Parthasarathy , “Noncardiogenic Pulmonary Edema and Life‐Threatening Shock due to Calcium Channel Blocker Overdose: A Case Report and Clinical Review,” Respiratory Care 59, no. 2 (2014): e15–e21. 23674813 10.4187/respcare.02244

[6] M. ST‐ONGE,1,2,3 P.‐A. DUB É , et al., “Treatment for Calcium Channel Blocker Poisoning: A Systematic Review,” Clinical Toxicology 52 (2014): 926–944. 25283255 10.3109/15563650.2014.965827 PMC4245158

[7] Z. Zhan , J. Chen , H. Zhou , et al., “Chronic Alcohol Consumption Aggravates Acute Kidney Injury Through Integrin β1/JNK Signaling,” Redox Biology 77 (2024): 103386, 10.1016/j.redox.2024.103386. 39378615 PMC11491727

[8] Z. V. Varga , C. Matyas , J. Paloczi , and P. Pacher , “Alcohol Misuse and Kidney Injury: Epidemiological Evidence and Potential Mechanisms,” Alcohol Research: Current Reviews 38, no. 2 (2017): 283–288. 28988579 10.35946/arcr.v38.2.10 PMC5513691

[9] A. Khwaja , “KDIGO Clinical Practice Guidelines for Acute Kidney Injury,” Nephron. Clinical Practice 120, no. 4 (2012): 179–184, 10.1159/000339789. 22890468

[10] M. D. Tapan Patel , M. D. David Tietze , N. Ankit , and M. D. Mehta , “Proceedings (Baylor University. Medical Center),” Amlodipine Overdose 26, no. 4 (2013): 410–411. 10.1080/08998280.2013.11929022 PMC3777093 24082424

[11] V. B. Kute , P. R. Shah , K. R. Goplani , M. R. Gumber , A. V. Vanikar , and H. L. Trivedi , “Successful Treatment of Refractory Hypotension, Noncardiogenic Pulmonary Edema and Acute Kidney Injury After an Overdose of Amlodipine,” Indian Journal of Critical Care Medicine 15, no. 3 (2011): 182–184. 22013313 10.4103/0972-5229.84901 PMC3190472

[12] C. J. Kapelios , G. Karamanakos , S. Liatis , et al., “Recurrent Episodes of Life‐Threatening Vasodilatory Shock Following Unintentional Intoxication With Amlodipine,” Hellenic Journal of Cardiology 58, no. 5 (2017): 369–371. 27986618 10.1016/j.hjc.2016.12.001

[13] O. Mark D and K. Daphne Harrington , “Etiology and Diagnosis of Prerenal Disease and Acute Tubular Necrosis in Acute Kidney Injury in Adults,” Acute Renal Tubular Necrosis 34, no. 3 (2024): 777–785.

[14] M. H. Rosner and M. D. Okusa , “Drug‐Associated Acute Renal Failure in the Intensive Care Unit,” in Clinical Nephrotoxins ‐ Renal Injury From Drugs and Chemicals, 3rd ed., ed. M. E. De Broe , G. A. Porter , W. M. Bennett , and G. Deray (Kluwer Academic Press, 2008).

[15] B. D. Rose , Pathophysiology of Renal Disease, 2nd ed. (McGraw‐Hill, 1987).

[16] Q. Yang , Y. Wang , H. Chen , et al., “Protective Activities of Scutellarin Against Alcohol‐Induced Acute Kidney Injury,” Chemistry & Biodiversity 19, no. 11 (2022): e202200254, 10.1002/cbdv.202200254. 36177678

